# Non-Hermitian Sensing in Photonics and Electronics: A Review

**DOI:** 10.3390/s22113977

**Published:** 2022-05-24

**Authors:** Martino De Carlo, Francesco De Leonardis, Richard A. Soref, Luigi Colatorti, Vittorio M. N. Passaro

**Affiliations:** 1 Photonics Research Group, Department of Electrical and Information Engineering, Politecnico di Bari, Via E. Orabona 4, 70125 Bari, Italy; francesco.deleonardis@poliba.it (F.D.L.); luigi.colatorti@poliba.it (L.C.); vittorio.passaro@poliba.it (V.M.N.P.); 2 Department of Engineering, University of Massachusetts at Boston, Boston, MA 02125, USA; richard.soref@umb.edu

**Keywords:** non-Hermitian Hamiltonians, PT symmetry, anti-PT symmetry, exceptional point, exceptional surface, quasi-PT symmetry

## Abstract

Recently, non-Hermitian Hamiltonians have gained a lot of interest, especially in optics and electronics. In particular, the existence of real eigenvalues of non-Hermitian systems has opened a wide set of possibilities, especially, but not only, for sensing applications, exploiting the physics of exceptional points. In particular, the square root dependence of the eigenvalue splitting on different design parameters, exhibited by 2 × 2 non-Hermitian Hamiltonian matrices at the exceptional point, paved the way to the integration of high-performance sensors. The square root dependence of the eigenfrequencies on the design parameters is the reason for a theoretically infinite sensitivity in the proximity of the exceptional point. Recently, higher-order exceptional points have demonstrated the possibility of achieving the *n*th root dependence of the eigenfrequency splitting on perturbations. However, the exceptional sensitivity to external parameters is, at the same time, the major drawback of non-Hermitian configurations, leading to the high influence of noise. In this review, the basic principles of PT-symmetric and anti-PT-symmetric Hamiltonians will be shown, both in photonics and in electronics. The influence of noise on non-Hermitian configurations will be investigated and the newest solutions to overcome these problems will be illustrated. Finally, an overview of the newest outstanding results in sensing applications of non-Hermitian photonics and electronics will be provided.

## 1. Introduction

In quantum mechanics, the Hamiltonian *Ĥ*, describing a closed quantum system, is a Hermitian operator (*Ĥ* = *Ĥ*^†^) [1]; it has real eigenvalues and orthogonal eigenstates, providing a complete basis in Hilbert space. Hermiticity guarantees the conservation of probability in an isolated quantum system [1].

During the 20th century, non-Hermitian Hamiltonians (*Ĥ* ≠ *Ĥ*^†^) were introduced to describe open systems [2]. Non-Hermitian Hamiltonians generally exhibit complex eigenvalues, and their eigenstates can be non-orthogonal. Non-Hermitian degeneracies happen at an exceptional point (EP) where two or more eigenvalues and the corresponding eigenstates coalesce simultaneously.

The widespread recent interest in non-Hermitian Hamiltonians takes its origin from the pioneering study by Bender et al. [3] in 1998. They demonstrated that a particular family of non-Hermitian Hamiltonians commuting with the joint operations of the parity operator (*P*) and time operator (*T*) ([*Ĥ*, *PT*] = 0) exhibit entirely real spectra under certain ranges of the design parameters, with non-orthogonal eigenstates. The properties of EPs of parity-time-symmetric Hamiltonians inspired lots of works, both in fundamental and in applied research, dealing with several fields of science, including optics [4,5,6,7,8], acoustics [9,10,11], electronics [12,13], metamaterials [14,15,16,17], spintronics [18,19] optomechanics [20], and others. Useful papers reviewing several applications of non-Hermitian systems in different fields can be found in [21,22,23].

Optics has been the most fertile branch of physics for the investigation of non-Hermitian Hamiltonians [4,5,6,7], starting from the work by Ruschhaupt et al. [24]: since the Schrödinger Hamiltonian is PT symmetric, provided that its potential (*V*) satisfies the condition *V*(**r**) = V*(−**r**) (with **r** the position vector), and since the refractive index plays the role of an optical potential in the spatial diffraction equation (isomorphic to the Schrödinger equation [25]), a refractive index with a symmetric real part and an antisymmetric imaginary part guarantees PT symmetry in optics. Later, in [26], a new class of synthetic photonic systems was investigated, with the antisymmetric refractive index (*n*(**r**) = −n*(−**r**)) under the joint *P* and *T* operators, defined as “antisymmetric parity-time (APT)”. An anti-PT-symmetric Hamiltonian (as it later has been called) satisfies the anticommutation relation {*Ĥ*, *PT*} = 0 [27] and can be realized without the necessity of gain.

About PT and anti-PT symmetries, a lot of research, both theoretical and experimental, has been carried out, especially in optics, dealing with power oscillations [28,29,30], PT-symmetric lasers [31,32], non-reciprocal optical propagation [33,34,35,36,37], unidirectional lasing [38,39], unidirectional invisibility [40,41], coherent-perfect absorption [42,43,44,45], electromagnetically induced transparency [46], orbital angular momentum lasers [47], nonlinear switching [48], nonlinear quantum spectroscopy [49], optomechanical actuation [50], optomechanical amplification [51], and magneto-optic isolation [52]. Useful reviews dealing with non-Hermitian photonics can be found in [4,5,6,7]. Among all the studied applications of non-Hermitian Hamiltonians, sensing is one of the most investigated. The reason for this interest relies upon the fact that at the EP, the eigenvalues of the system are extremely sensitive to applied perturbations. In particular, given a perturbation *ε* applied to the system, the eigenvalues (that coalesce at the EP) exhibit a square root dependence on the perturbation (*ε*^1/2^). This justifies the theoretically infinite sensitivity for very small perturbations, and the consequent interest for sensing applications.

Effective Hamiltonians with PT symmetry can be easily realized with resonators. In optics, PT-symmetric systems are usually realized through an active cavity and a passive cavity having the same resonant frequencies, and with perfectly balanced gain and loss.

It is intuitive that the same effective Hamiltonian can be easily reached in electronics (and not only) with RLC resonators, with perfectly balanced gain and loss (achieved via a resistor *R* and a perfectly matched negative resistor, −*R,* respectively).

In this review, an overview of the theory of non-Hermitian (PT-symmetric and anti-PT-symmetric) Hamiltonians in optics and electronics will be provided, with a focus on the most interesting recent applications related to sensing.

## 2. Non-Hermitian Hamiltonians

In quantum mechanics, the Hamiltonian, *Ĥ*, of a system governs the time evolution of the system itself, according to the Schrödinger-like equation, with

(1) $$i\frac{d}{dt}|\psi \rangle =\hat{H}|\psi \rangle ,$$

where |ψ〉 is the state vector of the system. The Hamiltonian of a system is an operator corresponding to the total energy of that system. According to the Dirac formalism, the spectrum of the allowed energy levels of the system in stationary conditions is given by the set of eigenvalues {*E*}, solving the equation

(2) $$\hat{H}|\psi \rangle =E|\psi \rangle .$$

In quantum mechanics, the Hamiltonian *Ĥ* is assumed to be Hermitian, *Ĥ* = *Ĥ*^†^ (the superscript † represents the Hermitian adjoint, i.e., transposition plus complex conjugation) [1]. The Hermiticity ensures that all the eigenvalues, *E,* are real and also guarantees a unitary time evolution [1].

There are several systems that can be described by a Schrödinger-like equation, including also a source term:

(3) $$i\frac{d}{dt}a=\hat{H}a+D,$$

where *a* is the amplitude vector and *D* a driving term. In [3], Bender et al. discovered that Hermiticity is not a necessary condition for *Ĥ* to have real eigenvalues. In particular, there exists a whole class of non-Hermitian Hamiltonians that shows real eigenvalues. This non-exclusive class has the property of being PT-symmetric.

## 3. Parity-Time Symmetry

A system is PT-symmetric provided that its Hamiltonian commutes with the PT operator ([*PT*, *Ĥ*] = 0), meaning that

(4) $$PT\hat{H}=\hat{H}PT,$$

and, consequently,

(5) $$PT\hat{H}{\left(PT\right)}^{-1}=\hat{H}.$$

**Figure 1:**
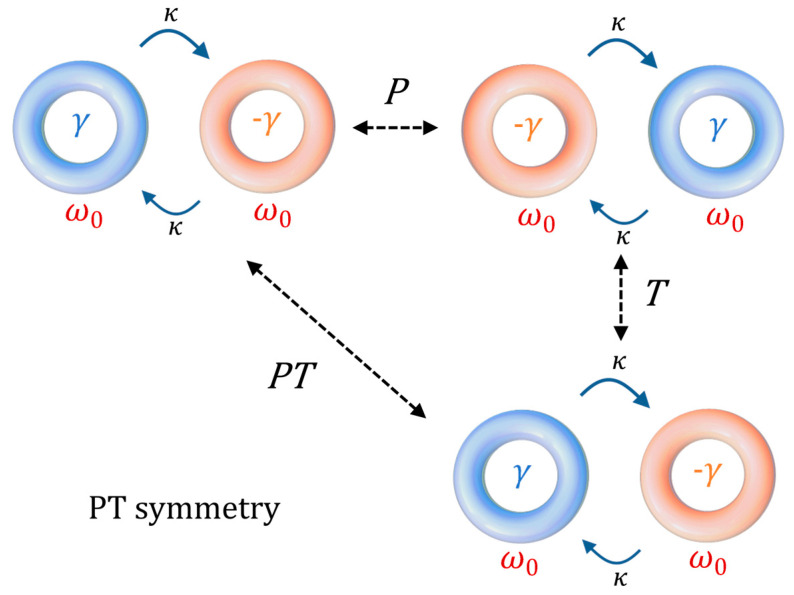
Graphical interpretation of PT symmetry in coupled resonators: γ represents the loss term, ω_0_ is the resonant frequency of each resonator, and κ is the coupling strength between the resonators. The system is invariant under simultaneous applications of the parity and time operations.

In other words, a Hamiltonian is PT-symmetric provided that it is invariant under the combined action of the *P* and *T* operations [3] (see Figure 1 for a graphical interpretation). In order to obtain the required condition for a Hamiltonian to be PT-symmetric, let us consider the parity operator, defined as

(6) $$P:\;\left(\begin{array}{c} x\\ y\\ z \end{array}\right)\to \left(\begin{array}{c} -x\\ -y\\ -z \end{array}\right)$$

and the time operator, defined as

(7) T: t→−t.

Let us now consider a simple 2 × 2 Hamiltonian, realized, for example, by coupling two generic resonators:

(8) $$\hat{H}=\left[\begin{array}{cc} -\omega_{c,1} & \kappa_{12}\\ \kappa_{21} & -\omega_{c,2} \end{array}\right]$$

where ω*_c_*_,1_ and ω*_c_*_,2_ are the complex resonances of two resonators (ω*_c_*_,1(2)_ = ω_1(2)_ + *i*γ_1(2)_), with ω_1(2)_ the real resonance frequency and γ_1(2)_ the decay rate in the resonator), and κ_12_ and κ_21_ are the coupling strengths between the two resonators.

In this context, the driving term *D* in (3) can be expressed as *D* = (*µ*_1_*s_in_*_1_, *µ*_2_*s_in_*_2_)^T^, where *s_in_*_1(2)_ is the input signal coupled to the first (second) resonator and *µ*_1(2)_ a coupling coefficient between *s_in_*_1(2)_ and the amplitude, a_1(2)_, in the first (second) resonator.

In (8), it has been implicitly considered that the time dependence is exp(*i*ω*t*). This choice differs from the one adopted by the majority of papers in physics, but it is useful for being consistent with the classical conventions adopted in optics and electronics (phasor notation) of the next sections.

Considering the matrix expression of the Hamiltonian in (7), the parity operator is the Pauli operator:

(9) $$P=\left[\begin{array}{cc} 0 & 1\\ 1 & 0 \end{array}\right]$$

and the *T* operator acts on the operators as

(10) $$T\hat{H}T^{-1}={\hat{H}}^{*}.$$

So, to verify the condition necessary for the PT symmetry:

(11) $$PT\hat{H}{\left(PT\right)}^{-1}=\left[\begin{array}{cc} 0 & 1\\ 1 & 0 \end{array}\right]\left[\begin{array}{cc} -\omega_{c,1}^{*} & \kappa_{12}^{*}\\ \kappa_{21}^{*} & -\omega_{c,2}^{*} \end{array}\right]\left[\begin{array}{cc} 0 & 1\\ 1 & 0 \end{array}\right]=\left[\begin{array}{cc} -\omega_{c,2}^{*} & \kappa_{21}^{*}\\ \kappa_{12}^{*} & -\omega_{c,1}^{*} \end{array}\right]$$

It is immediately seen that the required condition for a 2 × 2 Hamiltonian to be PT-symmetric (see Equation (5)) are:

(12) $$\omega_{c,1}=\omega_{c,2}^{*}=\omega_{0}+i\gamma$$

(13) $$\kappa_{12}=\kappa_{21}^{*}.$$

For reciprocal coupling mechanisms (κ_12_ = κ_21_ = κ), the second requirement is equivalent to have κ be a real number. So, the PT-symmetric Hamiltonian is found to be

(14) $${\hat{H}}_{PT}=\left[\begin{array}{cc} -\omega_{0}-i\gamma & \kappa \\ \kappa & -\omega_{0}+i\gamma \end{array}\right]$$

where ω_0_, γ and κ are real values. For two coupled resonators, ω_0_ is the same resonant frequency for the two resonators, κ is the coupling strength between the resonators, and γ is the loss term (−γ can be seen as the linear gain; in our model we will neglect the effect of gain saturation).

The set of eigenvalues {−ω*_PT_*} of the Hamiltonian is easily found by setting

(15) $$\det\left[\begin{array}{cc} -\omega_{0}-i\gamma +\omega_{PT} & \kappa \\ \kappa & -\omega_{0}+i\gamma +\omega_{PT} \end{array}\right]=0.$$

So, we obtain

(16) $$\omega_{PT}=\omega_{0}\pm \sqrt{\kappa^{2}-\gamma^{2}}.$$

The two eigenfrequencies found can be designed to coalesce, for |κ| = |γ|, in ω*_PT_* = ω_0_. This design condition is called the “exceptional point” (EP). The exceptionality of this design condition arises from the fact that, as soon as a perturbation, ε, is applied to any of the parameters of the system (resonances, gain or loss of one resonator, coupling strength), the two eigenfrequencies split according to a square root function of the perturbation. In particular, there are three possible kinds of perturbation that can be interesting for sensing purposes:

- perturbation of the resonances of each cavity;
- perturbation of the loss (gain) of each cavity;
- perturbation of the coupling mechanism between the cavities.

For |κ| > |γ|, the PT symmetry is called “unbroken” (exhibiting real eigenvalues), whereas, for |κ| < |γ|, the PT symmetry is called “broken” (exhibiting complex conjugate eigenvalues). In the unbroken PT symmetry, bifurcating eigenmodes appear. The eigenmodes oscillate and do not grow or decay Instead, in broken PT symmetry, the system is not in equilibrium; one eigenmode grows in time and the other decays in time.

### 3.1. Perturbing Resonances in PT Symmetry

When a perturbation ε_ω_ is applied to one of the resonances of a PT-symmetric Hamiltonian (in the following, we will consider the first of the two resonances being perturbed), the new Hamiltonian becomes

(17) $${\hat{H}}_{PT,\varepsilon_{\omega }}={\hat{H}}_{PT}+{\hat{H}}_{\varepsilon_{\omega }}=\left[\begin{array}{cc} -\omega_{0}-i\gamma -\varepsilon_{\omega } & \kappa \\ \kappa & -\omega_{0}+i\gamma \end{array}\right].$$

The eigenfrequencies become

(18) $$\omega_{PT,\varepsilon_{\omega }}=\omega_{0}+\varepsilon_{\omega }/2\pm \sqrt{\kappa^{2}-{\left(\gamma -i\varepsilon_{\omega }/2\right)}^{2}}.$$

With a design at the EP (|κ| = |γ|) and with ε_ω_ << |κ|:

(19) $$\omega_{PT,\varepsilon_{\omega }}\approx \omega_{0}+\varepsilon_{\omega }/2\pm \frac{1+i}{\sqrt{2}}\sqrt{{\kappa \varepsilon }_{\omega }}.$$

The result is that the splitting between the eigenfrequencies is proportional to the square root of the perturbation. The sensitivity of the eigenfrequencies splitting to the perturbation ε_ω_ at the EP is proportional to the inverse of the square root of the perturbation (ε_ω_^−1/2^), thus leading to an infinite sensitivity for ε_ω_ tending to zero.

### 3.2. Perturbing Loss (Gain) in PT Symmetry

When a perturbation ε_γ_ is applied to the loss (gain) of one of the resonators (in the following, we will consider the first of the two resonators being perturbed), the new Hamiltonian becomes

(20) $${\hat{H}}_{PT,\varepsilon_{\gamma }}={\hat{H}}_{PT}+{\hat{H}}_{\varepsilon_{\gamma }}=\left[\begin{array}{cc} -\omega_{0}-i\gamma -i\varepsilon_{\gamma } & \kappa \\ \kappa & -\omega_{0}+i\gamma \end{array}\right].$$

The eigenfrequencies become

(21) $$\omega_{PT,\varepsilon_{\gamma }}=\omega_{0}+i\varepsilon_{\gamma }/2\pm \sqrt{\kappa^{2}-{\left(\gamma +\varepsilon_{\gamma }/2\right)}^{2}}.$$

With a design at the EP (|κ| = |γ|) and with ε_γ_ << |κ|:

(22) $$\omega_{PT,\varepsilon_{\gamma }}\approx \omega_{0}+i\varepsilon_{\gamma }/2\pm i\sqrt{{\kappa \varepsilon }_{\gamma }}.$$

The result is that the splitting between the eigenfrequencies is proportional to the square root of the perturbation and the sensitivity of the eigenfrequency splitting to the perturbation is infinite for ε_γ_ tending to zero.

**Figure 2:**
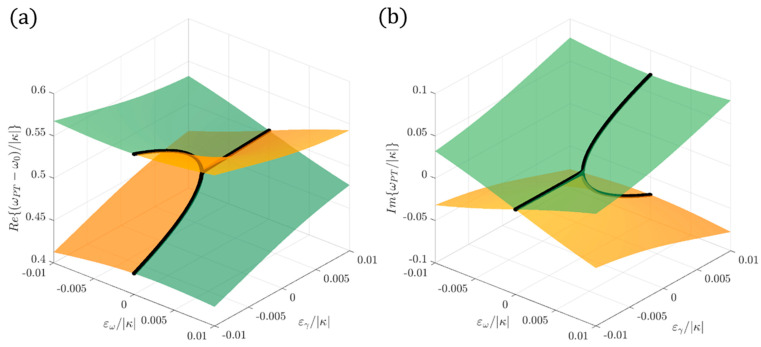
The real part (**a**) and imaginary part (**b**) of the eigenfrequencies of a PT-symmetric Hamiltonian for different values of the perturbations ε_ω_ and ε_γ_ in the proximity of an EP. Black lines identify the region where ε_ω_ = 0.

Figure 2 shows the real part (Figure 2a) and the imaginary part (Figure 2b) of the eigenfrequencies of a PT-symmetric Hamiltonian for different values of the perturbations ε_ω_ and ε_γ_ in the proximity of an EP. Black lines identify the region where ε_ω_ = 0: it is possible to appreciate the square root dependence on ε_γ_ in the proximity of the EP.

### 3.3. Perturbing Coupling Strength in PT Symmetry

When a perturbation ε_κ_ is applied to the coupling mechanism between the resonators (the coupling is supposed to be reciprocal), the new Hamiltonian becomes

(23) $${\hat{H}}_{PT,\varepsilon_{\kappa }}={\hat{H}}_{PT}+{\hat{H}}_{\varepsilon_{\kappa }}=\left[\begin{array}{cc} -\omega_{0}-i\gamma & \kappa \;+\varepsilon_{\kappa }\\ \kappa +\varepsilon_{\kappa } & -\omega_{0}+i\gamma \end{array}\right].$$

The eigenfrequencies become

(24) $$\omega_{PT,\varepsilon_{\kappa }}=\omega_{0}\pm \sqrt{{\left(\kappa +\varepsilon_{\kappa }\right)}^{2}-\gamma^{2}}.$$

With a design at the EP (|κ| = |γ|) and with ε_κ_ << |κ|:

(25) $$\omega_{PT,\varepsilon_{\kappa }}\approx \omega_{0}\pm \sqrt{2{\kappa \varepsilon }_{\kappa }}.$$

The result is that the splitting between the eigenfrequencies is proportional to the square root of the perturbation and the sensitivity is proportional to the inverse of the square root of the perturbation.

## 4. Anti-Parity-Time Symmetry

A system is anti-PT-symmetric, provided that its Hamiltonian satisfies the anticommutation relation with the *PT* operator ({*PT*, *Ĥ*} = 0), meaning that:

(26) $$PT\hat{H}{\left(PT\right)}^{-1}=-\hat{H}.$$

**Figure 3:**
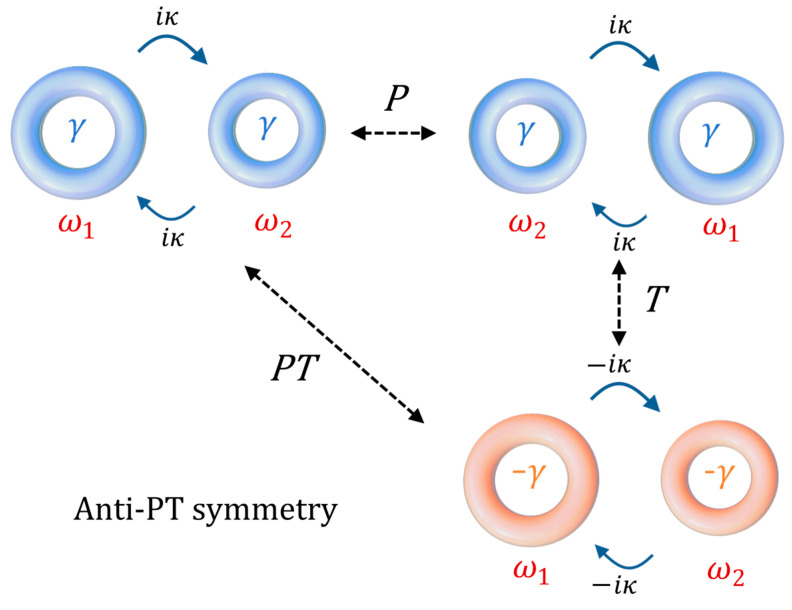
Graphical interpretation of anti PT symmetry in coupled resonators.

In other words, under the combined action of the *P* and *T* operations [27], the obtained Hamiltonian is the opposite of the starting one (see Figure 3 for a graphical interpretation).

To find the conditions required for a Hamiltonian to be anti-PT-symmetric, let us apply the definition:

(27) $$PT\hat{H}{\left(PT\right)}^{-1}=\left[\begin{array}{cc} 0 & 1\\ 1 & 0 \end{array}\right]\left[\begin{array}{cc} -\omega_{c,1}^{*} & \kappa_{12}^{*}\\ \kappa_{21}^{*} & -\omega_{c,2}^{*} \end{array}\right]\left[\begin{array}{cc} 0 & 1\\ 1 & 0 \end{array}\right]=\left[\begin{array}{cc} -\omega_{c,2}^{*} & \kappa_{21}^{*}\\ \kappa_{12}^{*} & -\omega_{c,1}^{*} \end{array}\right].$$

It is immediately seen that, in order to be anti-PT-symmetric (see Equation (26)), a Hamiltonian requires:

(28) $$\omega_{c,1}=-\omega_{c,2}^{*}=\Delta +i\gamma$$

(29) $$\kappa_{12}=-\kappa_{21}^{*}$$

For reciprocal coupling mechanisms (κ_12_ = κ_21_), the second requirement is equivalent to having an imaginary coupling strength. So, the anti-PT-symmetric Hamiltonian is found to be

(30) $${\hat{H}}_{APT}=\left[\begin{array}{cc} -\Delta -i\gamma & i\kappa \\ i\kappa & \Delta -i\gamma \end{array}\right]$$

where Δ, γ, and κ are real values.

However, this condition is not realistically satisfiable by two coupled resonators, because it would require having at least one negative resonance frequency (without a physical meaning). Nonetheless, a Hamiltonian, *Ĥ_QAPT_*, describing two coupled resonators with different resonances (ω_1_ and ω_2_), the same loss (or gain), and imaginary coupling strength, can be rewritten in the form of an anti-PT-symmetric Hamiltonian after transforming the equation of motion to the frame rotating with the carrier frequency ω_0_ (with ω_0_ = (ω_1_ + ω_2_)/2). Starting from the Schrödinger-like equation:

(31) $$i\frac{d}{dt}\left[\begin{array}{c} A_{1}\\ A_{2} \end{array}\right]=\left[\begin{array}{cc} -\omega_{1}-i\gamma & i\kappa \\ i\kappa & -\omega_{2}-i\gamma \end{array}\right]\left[\begin{array}{c} A_{1}\\ A_{2} \end{array}\right]={\hat{H}}_{QAPT}\left[\begin{array}{c} A_{1}\\ A_{2} \end{array}\right],$$

where (*A*_1_, *A*_2_)^T^ is the amplitude vector, and applying the transformation to the rotating frame,

(32) $$\left[\begin{array}{c} A_{1}^{\prime }\\ A_{2}^{\prime } \end{array}\right]=e^{-i\omega_{0}t}\left[\begin{array}{c} A_{1}\\ A_{2} \end{array}\right],$$

we obtain

(33) $$i\frac{d}{dt}\left[\begin{array}{c} A_{1}^{\prime }\\ A_{2}^{\prime } \end{array}\right]=\left[\begin{array}{cc} -\Delta +i\gamma & i\kappa \\ i\kappa & \Delta +i\gamma \end{array}\right]\left[\begin{array}{c} A_{1}^{\prime }\\ A_{2}^{\prime } \end{array}\right]$$

with ($A_{1}^{\prime }$, $A_{2}^{\prime }$)^T^ the state vector in the rotating frame and Δ = (ω_1_ − ω_2_)/2. Since rotating the frequency frame of reference does not affect the properties of the eigenfrequencies, we can continue to refer to *Ĥ_QAPT_* as an anti-PT-symmetric Hamiltonian. This is the reason why the configuration in Figure 3 is referred as anti-PT symmetric.

The set of eigenvalues of *Ĥ_QAPT_* and *Ĥ_APT_* will only differ by ω_0_. Referring to *Ĥ_QAPT_*, the set of the eigenvalues {−ω*_APT_*} is easily found by setting

(34) $$\det\left[\begin{array}{cc} -\omega_{1}-i\gamma +\omega_{APT} & i\kappa \\ i\kappa & -\omega_{2}-i\gamma +\omega_{APT} \end{array}\right]=0$$

So, we obtain

(35) $$\omega_{APT}=\omega_{0}+i\gamma \pm \sqrt{\Delta^{2}-\kappa^{2}}.$$

The two eigenfrequencies found can be designed to coalesce, for |κ| = |Δ|, in ω*_APT_* = ω_0_. This design condition corresponds to the EP. The exceptionality of this design condition arises from the fact that, as soon a perturbation, ε, is applied to any of the parameters of the involved system (isolated resonances, gain or loss of one resonator, coupling strength), the two eigenfrequencies split according to a square root function of the perturbation. For |Δ| < |κ|, the anti-PT symmetry is called “unbroken”, whereas, for |Δ| > |κ|, the anti-PT symmetry is called “broken”. In the unbroken anti-PT symmetry, the eigenmodes have the same resonance frequency but different linewidths. Instead, in broken anti-PT symmetry, bifurcating eigenmodes appear (with distinguishable resonant peaks).

### 4.1. Perturbing Resonances in Anti-PT Symmetry

When a perturbation ε_ω_ is applied to one of the resonances of an anti-PT-symmetric Hamiltonian (in the following, we will consider the first of the two resonances being perturbed), the new Hamiltonian becomes

(36) $${\hat{H}}_{QAPT,\varepsilon_{\omega }}={\hat{H}}_{QAPT}+{\hat{H}}_{\varepsilon_{\omega }}=\left[\begin{array}{cc} -\omega_{1}-i\gamma -\varepsilon_{\omega } & i\kappa \\ i\kappa & -\omega_{2}-i\gamma \end{array}\right].$$

The eigenfrequencies become

(37) $$\omega_{APT,\varepsilon_{\omega }}=\omega_{0}+\varepsilon_{\omega }/2+i\gamma \pm \sqrt{{\left(\Delta +\varepsilon_{\omega }/2\right)}^{2}-\kappa^{2}}.$$

Without loss of generality, in the simplifying hypothesis of ω_1_ > ω_2_ and with a design at the EP (|κ| = |Δ|), and with ε_ω_ << |κ|:

(38) $$\omega_{APT,\varepsilon_{\omega }}\approx \omega_{0}+\varepsilon_{\omega }/2+i\gamma \pm \sqrt{{\kappa \varepsilon }_{\omega }}.$$

The result is that the splitting between the eigenfrequencies is proportional to the square root of the perturbation.

### 4.2. Perturbing Loss (Gain) in Anti-PT Symmetry

When a perturbation ε_γ_ is applied to the loss (gain) of one of the resonators (in the following, we will consider the first of the two resonators being perturbed), the new Hamiltonian becomes

(39) $${\hat{H}}_{QAPT,\varepsilon_{\gamma }}={\hat{H}}_{QAPT}+{\hat{H}}_{\varepsilon_{\gamma }}=\left[\begin{array}{cc} -\omega_{1}-i\gamma -i\varepsilon_{\gamma } & i\kappa \\ i\kappa & -\omega_{2}-i\gamma \end{array}\right].$$

The eigenfrequencies become

(40) $$\omega_{APT,\varepsilon_{\gamma }}=\omega_{0}+i\varepsilon_{\gamma }/2+i\gamma \pm \sqrt{{\left(\Delta +i\varepsilon_{\gamma }/2\right)}^{2}-\kappa^{2}}.$$

Without loss of generality, in the simplifying hypothesis of ω_1_ > ω_2,_ with a design at the EP (|κ| = |Δ|), and with ε_γ_ << |κ|:

(41) $$\omega_{APT,\varepsilon_{\gamma }}\approx \omega_{0}+i\varepsilon_{\gamma }/2+i\gamma \pm \frac{1+i}{\sqrt{2}}\sqrt{{\kappa \varepsilon }_{\gamma }}.$$

The result is that the splitting between the eigenfrequencies is proportional to the square root of the perturbation.

**Figure 4:**
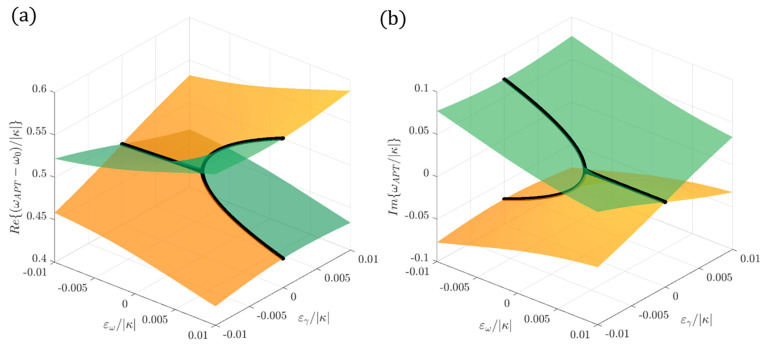
The real part (**a**) and imaginary part (**b**) of the eigenfrequencies of an anti-PT-symmetric Hamiltonian for different values of the perturbations ε_ω_ and ε_γ_ in the proximity of an EP. Black lines identify the region where ε_γ_ = 0.

Figure 4 shows the real part (Figure 4a) and the imaginary part (Figure 4b) of the eigenfrequencies of an anti-PT-symmetric Hamiltonian for different values of the perturbations ε_ω_ and ε_γ_ in the proximity of an EP. Black lines identify the region where ε_γ_ = 0: it is possible to appreciate the square root dependence on ε_ω_ in the proximity of the EP.

### 4.3. Perturbing Coupling Mechanism in Anti-PT Symmetry

When a perturbation, ε_κ_, is applied to the coupling mechanism between the resonators (the coupling mechanism is supposed to be reciprocal), the new Hamiltonian becomes

(42) $${\hat{H}}_{QAPT,\varepsilon_{\kappa }}={\hat{H}}_{QAPT}+{\hat{H}}_{\varepsilon_{\kappa }}=\left[\begin{array}{cc} -\omega_{1}-i\gamma & i\kappa +\varepsilon_{\kappa }\\ i\kappa +\varepsilon_{\kappa } & -\omega_{2}-i\gamma \end{array}\right].$$

The eigenfrequencies become

(43) $$\omega_{APT,\varepsilon_{\kappa }}=\omega_{0}+i\gamma \pm \sqrt{\Delta^{2}-{\left(\kappa +\varepsilon_{\kappa }\right)}^{2}}.$$

So, we obtain, with a design at the EP (|κ| = |Δ|), and with ε_κ_ << |κ|:

(44) $$\omega_{APT,\varepsilon_{\kappa }}\approx \omega_{0}+i\gamma \pm i\sqrt{2{\kappa \varepsilon }_{\kappa }}.$$

The result is that the splitting between the eigenfrequencies is proportional to the square root of the perturbation.

## 5. Stability and Noise in Non-Hermitian Hamiltonians

By definition, the PT-symmetric Hamiltonian is designed to work with the eigenfrequencies at the limit of stability. In fact, the time behaviour of the eigenmodes in the cavities can be easily obtained by using the found eigenfrequencies. An eigenfrequency $\omega_{A}$ corresponds to an eigenmode $E_{A}$, such that [53]

(45) $$E_{A}\propto e^{j\omega_{A}t}.$$

The immediate consequence is that a negative imaginary part of an eigenfrequency leads to a divergent eigenmode. So, a PT-symmetric system at the EP, by its definition, is at the limit of stability (normally stable), because the coalesced eigenfrequencies in the unperturbed condition lie on the real axis. Any source of noise would make the system exit the stability condition, leading to the presence of divergent eigenmodes, and lasing would arise. Instead, by its definition, anti-PT symmetry at the EP can be stable, provided that γ > 0 (system not lasing).

**Figure 5:**
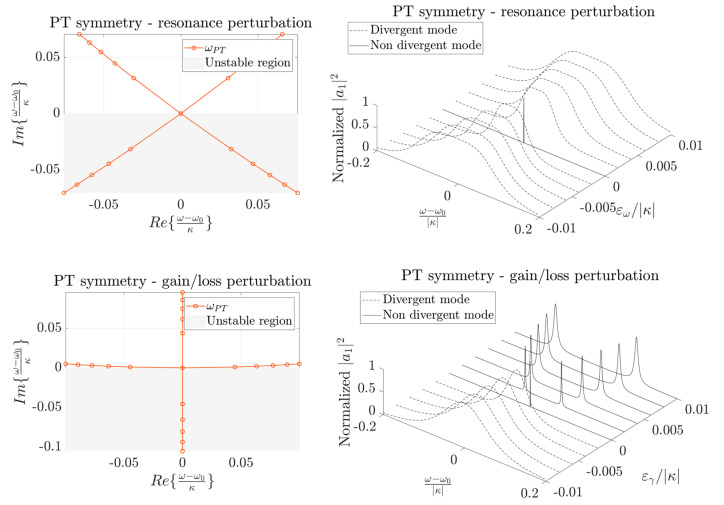

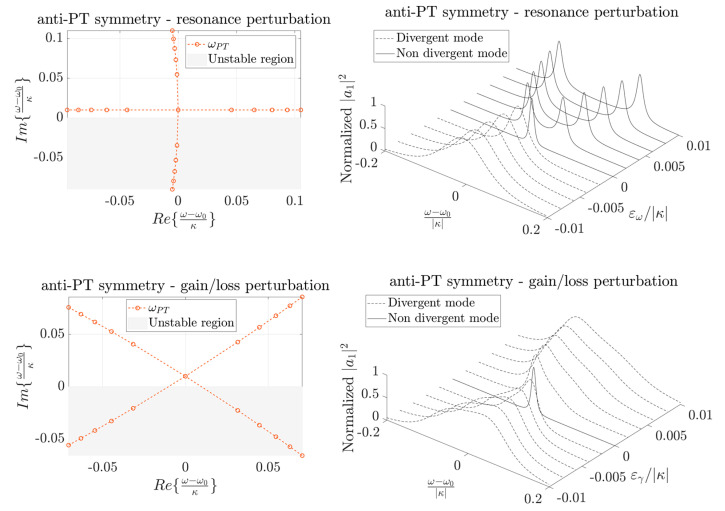
Eigenfrequency trajectories in the Gauss plane for PT-symmetric and anti-PT-symmetric systems (**left column**) due to perturbations of the resonance frequency or of the loss (gain) of one resonator: eigenfrequencies falling in the grey region (unstable region) causes instability. Normalized energy in one of the resonators (**right column**) of PT-symmetric and anti-PT-symmetric systems (considering inputs *s_in1_* = 1 and *s_in2_* = 0) as a function of the value of the perturbation of the resonance (*ε*_ω_), or of the loss (gain) (*ε*_γ_): solid lines represent stable transfer functions and dashed lines represent unstable transfer functions.

Figure 5, on the left column, shows the trajectories of the eigenfrequencies on the Gauss plane (Re{ω}, Im{ω}) for PT-symmetric and anti-PT-symmetric configurations in the presence of the perturbation of the resonance or of the gain (or loss) of one of the resonators. The grey half-plane represents the unstable region, i.e., the region where eigenfrequencies should not lie in order to prevent instability. As soon as one eigenfrequency lies in the unstable plane, the system becomes unstable, due to the presence of at least one divergent mode. The right column of Figure 5 shows the normalized energy in one of the resonators (proportional to a measurable output of the system) as a function of the normalized angular frequency and for the same values of the perturbations used in the corresponding graph on the left column. To obtain the normalized graph in the right column of Figure 5, input amplitudes *s_in_*_1_ = 1 and *s_in_*_2_ = 0 have been considered.

Since the imaginary part of an eigenfrequency is proportional to the linewidth of the corresponding eigenmode, the fact that the eigenfrequencies of the PT-symmetric case lie on the real axis of the Gauss plane is an advantage for the resolution of the sensor. However, as seen, the proximity of the eigenfrequency with the half plane, with Im{ω} < 0, leads to instability.

To overcome the problem of stability of PT symmetry, the concept of quasi-PT symmetry can be introduced. Quasi-PT symmetry can be useful also for implementing EP when introducing gain is not possible.

## 6. Quasi-PT Symmetry

Sometimes PT symmetry can be difficult to achieve because of the necessity of implementing gain. Moreover, it shows some problems of instability due to the presence of eigenfrequencies on the real axis of the complex plane. Several works [54,55] solved this issue by applying differential losses to the optical modes (*A*_1_ and *A*_2_) involved in the Hamiltonian. Let us consider the Schrödinger-like equation:

(46) $$i\frac{d}{dt}\left[\begin{array}{c} A_{1}\\ A_{2} \end{array}\right]=\left[\begin{array}{cc} -\omega_{0}-i\gamma_{1} & \kappa \\ \kappa & -\omega_{0}-i\gamma_{2} \end{array}\right]\left[\begin{array}{c} A_{1}\\ A_{2} \end{array}\right].$$

We can use a variable transformation to introduce two auxiliary modes that experience a common gain with respect to *A*_1_ and *A*_2_:

(47) $$\left[\begin{array}{c} {\tilde{A}}_{1}\\ {\tilde{A}}_{2} \end{array}\right]=e^{\;\chi t}\left[\begin{array}{c} A_{1}\\ A_{2} \end{array}\right]$$

where *χ* = (γ_1_ + γ_2_)/2. In this way, a new Hamiltonian can be introduced, such that

(48) $$i\frac{d}{dt}\left[\begin{array}{c} {\tilde{A}}_{1}\\ {\tilde{A}}_{2} \end{array}\right]=\left[\begin{array}{cc} -\omega_{0}-i\xi & \kappa \\ \kappa & -\omega_{0}+i\xi \end{array}\right]\left[\begin{array}{c} {\tilde{A}}_{1}\\ {\tilde{A}}_{2} \end{array}\right]$$

where *ξ* = (γ_1_ − γ_2_)/2.

In this way, the PT symmetry is verified. An EP still exists. This common practice, however, is different from the similar variable change applied in the anti-PT-symmetric case and has some drawbacks in the measurable outputs. In fact, a common loss can spoil the resolvability of the resonances, due to the broadening of the linewidths (see [56]).

## 7. Noise and Limit of Detection in Non-Hermitian Hamiltonians

### 7.1. Classical Noise in Non-Hermitian Hamiltonians

The incredibly enhanced sensitivity to target parameters of non-Hermitian Hamiltonians has its immediate drawback in the enhanced sensitivity also to unwanted perturbation and noise.

The influence of classical noise has been investigated in [21]. Including noise in the total non-Hermitian Hamiltonian of a system; i.e., considering a noisy EP, it is possible to obtain

(49) $${\hat{H}}_{\mathrm{tot}}\left(t\right)=\hat{H}\left(\varepsilon \right)+\overset{K}{\underset{j=1}{\sum }}\xi_{j}\left(t\right){\hat{H}}_{\mathrm{noise},j}$$

where *K* is the number of statistically independent noise sources and the *ξ_j_* are real-valued fluctuations with a zero mean, whereas *Ĥ*_noise,j_ describe the fluctuations of the matrix elements of the total Hamiltonian.

Several papers have studied the influence of noise on the EP. In [57], the authors investigated the influence of mesoscopic fluctuations and noise on the spectral and temporal properties of systems of PT-symmetric-coupled gain-loss resonators at the EP. By considering an inevitable detuning of the resonance frequencies of the isolated resonators, the authors obtain that statistical averaging significantly smears the spectral features, thus limiting the sensitivity of EP-based sensors. Moreover, they showed that temporal fluctuations in the resonance detuning and gain lead to a quadratic growth of the optical power in time, meaning dynamical instability.

The numerical simulations in [58] showed an exponential divergence of the eigenstates due to the presence of noise. The authors say that maintaining operation at the EP for enough time to detect resonance splitting requires very careful design of a feedback system. Nonlinearities that could prevent divergence are not included in the modelling.

In practice, for EP-based sensors operating close to the real frequency axis (as it happens in the PT-symmetric case), even a small noise can lead to instability, thus spoiling the resolvability. This does not mean that EP-based sensors are fundamentally limited by classical noise [21].

The instability can be removed, for example, by uniform damping of the sensor, thus realizing a quasi-PT-symmetric version of the sensor. However, as said, this would broaden the linewidths [56], thus reducing the resolution.

### 7.2. Quantum Noise

Different from classical noise, quantum noise may fundamentally limit EP-based sensing [59,60,61,62]. The theoretical approaches to analyse the quantum noise have been developed from the hypothesis of a weak dispersive limit, where the frequency splitting is much lower than the linewidths [21]. However, most of the experimental works have been performed away from this condition. The problem raised in the quantum noise studies about non-Hermitian systems arises from the fact that the frequency splitting is not a direct measurement but derives from measurement of the fields. In [59], it has been demonstrated that the changes in the fields in lowest order are proportional to the perturbation, both at the diabolical points [63] (where the splitting of the resonances is proportional to the perturbation) and at the EP. This would imply an equal scaling in the quantum-limited signal-to-noise ratio for EPs and diabolic points.

The same conclusion has been reached in [60], where it is demonstrated that an upper bound of the signal-to-noise ratio is the same independent of whether the sensor is at an EP or not. The bound obtained in [60], however, is only limited to reciprocal sensors. Non-reciprocity is seen as a good source to be exploited for higher performance sensing (see Section 8).

In [61], the signal-to-noise ratio bound in the quantum regime has been demonstrated to be better for an EP-based sensor near its lasing threshold, using a linearization approach (that could represent a limit in their analysis).

In [62], the authors observed that the diverging frequency splitting enhancement of a Brillouin-based optical gyroscope at the EP is exactly compensated by a diverging broadening of the laser linewidths, due to the non-orthogonality of the counterpropagating modes. The factor of the linewidth broadening is called the Petermann factor and is due to the coalescence of the eigenmodes at the EP.

The topic of the noise at the EP is still an open issue; so, a lot of research has been performed to realize sensors able to prevent the destructive effect of noise (see Section 8, Section 11 and Section 12.

### 7.3. Limit of Detection

The strong spectral response of PT- and anti-PT-symmetric Hamiltonians is expressed in the proximity of EPs. Far from these operating conditions, the systems behave like diabolic points.

So, since it is crucial to have a design in the proximity of the EP, we need the radicands in Equations (16) and (35) to be exactly at the EP.

In order to be able to detect a perturbation ε (applied for example to the loss or gain or to the resonance), in the proximity of an EP, we need that

(50) $$\left|\kappa \varepsilon \right|\gg \left|\delta_{EP}\right|,$$

where

(51) $$\{\begin{array}{cc} \delta_{EP}=\kappa^{2}-\gamma^{2} & \mathrm{for}\;a\;\mathrm{PT}-\mathrm{symmetric}\;\mathrm{configuration}\\ \delta_{EP}=\Delta^{2}-\kappa^{2} & \mathrm{for}\;\mathrm{an}\;\mathrm{anti}-\mathrm{PT}-\mathrm{symmetric}\;\mathrm{configuration} \end{array}$$

So, the limit of detection of the sensor is defined by the possibility of keeping the system at the EP, with the aid of a feedback loop.

## 8. Exceptional Surface

As demonstrated, in PT-symmetric implementation, the resonant frequencies of the two resonators need to be identical and the gain and loss need to be perfectly balanced. Finally, the coupling strength between the resonators needs to perfectly match the difference between gain and loss. Instead, in an anti-PT-symmetric device, the gains or losses of the two resonators need to be the same and the indirect coupling strength needs to match the difference between the isolated resonances.

Several researchers make use of feedback techniques to tune the system actively and continuously, setting it in the proximity of the EP (using micro-heaters, tuneable coupling methods, etc.). However, in this way the resolution of the sensor needs to be aligned to the resolution of the transducer of the active control. Moreover, it would be extremely useful for practical sensing application to propose a new design to decouple unwanted fabrication errors and experimental uncertainties from the target perturbations caused by the sensing mechanism.

Therefore, in [64], Zhong et al. proposed the idea of a hypersurface of EPs, called exceptional surface (ES). The idea is to make the condition of the EP insensitive to perturbations that are not related to the sensing principle.

**Figure 6:**
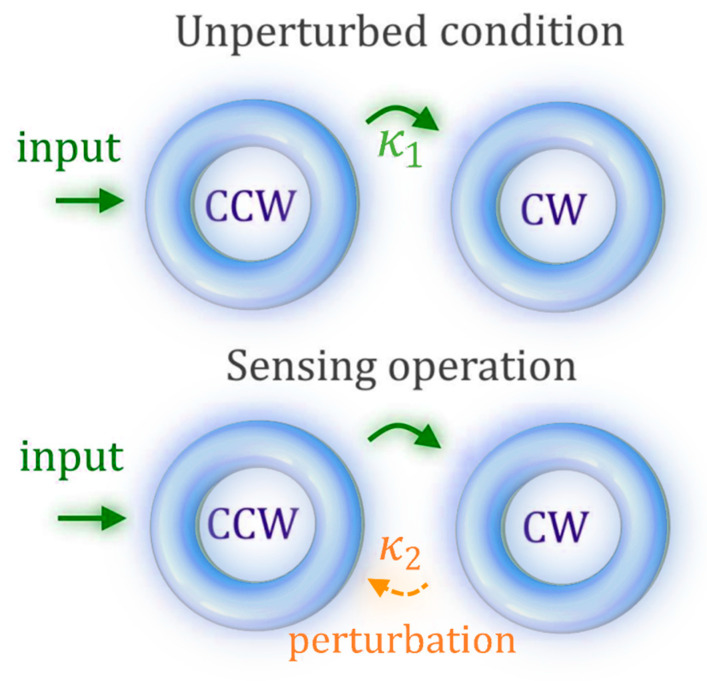
Exceptional surface-based configuration, illustrating schematically two non-reciprocally coupled modes within one optical resonator.

This idea is achieved by coupling two counterpropagating optical modes inside the same cavity (as in Figure 6), rather than using two separated coupled cavities. The architecture in Figure 6 can be described with the effective Hamiltonian *H_ES_*:

(52) $$i\frac{d}{dt}\left[\begin{array}{c} a_{cw}\\ a_{ccw} \end{array}\right]={\hat{H}}_{ES}\left[\begin{array}{c} a_{cw}\\ a_{ccw} \end{array}\right],\;H_{ES}=\left[\begin{array}{cc} -\omega_{0}-i\gamma & \kappa_{1}\\ \kappa_{2} & -\omega_{0}-i\gamma \end{array}\right],$$

where $a_{cw}$ and $a_{ccw}$ are the field amplitudes of the clockwise (CW) and counterclockwise (CCW) modes, $\omega_{0}$ is the optical resonance frequency of the optical cavity (same for CW and CCW modes), γ is the common loss per time unit, and $\kappa_{1}$ ($\kappa_{2}$) is the coupling strength between the CCW and the CW modes (CW and CCW).

The eigenfrequencies of the system are easily found in the harmonic regime:

(53) $${\omega_{ES}}_{1/2}=\omega_{0}+i\gamma \pm \sqrt{\kappa_{1}\kappa_{2}},$$

The main result, in this case, is that the EP is achieved when one of the two coupling strengths, $\kappa_{1}$ or $\kappa_{2}$, is equal to zero. In this case, any perturbation to the resonance $\omega_{0}$ or to the gain γ does not perturb the EP. Thus, there is a hypersurface of EPs, which can be called an exceptional surface (ES). For κ_1_ equal to zero, the eigenfrequencies difference depend on the square root of κ_2_, thus representing an important advantage for sensing. Figure 6 illustrates schematically the sensing principle.

However, the proposed architecture is not as versatile as the parity-time and anti-parity-time-symmetric systems presented before. This kind of configuration is useful for sensing principles only when the perturbations on the coupling strength represent the target of the sensing.

The concept of an exceptional surface has been recently investigated in optics in several research works [65,66,67,68,69].

## 9. Non-Hermitian Photonics

### 9.1. PT-Symmetric Optical Potential

Non-Hermitian Hamiltonians have been studied and developed especially in optics. The first demonstration of the possibility of realizing PT symmetry in optics was done in [24]. Then, a parallelism between the potential in the Schrödinger equation and the refractive index made it possible to conceptualize a new variety of optical PT-symmetric Hamiltonians.

The paraxial equation of diffraction in optics is [7]

(54) $$i\frac{dE\left(x,z\right)}{dz}+\frac{1}{2k}\frac{d^{2}E\left(x,z\right)}{dx^{2}}+k_{0}\left[n_{r}\left(x\right)+in_{i}\left(x\right)\right]E\left(x,z\right)=0,$$

where *E*(*x*,*z*) is the electric field envelope, *n_r_*(*x*) and *n_i_*(*x*) are the real and the imaginary part of the refractive index distribution, respectively, and *k*_0_ = 2π *n*_0_ /λ, with λ the wavelength of the field in vacuum and *n*_0_ the substrate index. This equation is widely known to be mathematically isomorphic to the Schrödinger equation:

(55) $$iћ\frac{d\psi \left(x,t\right)}{dt}+\frac{{ћ}^{2}}{2m}\frac{d^{2}\psi \left(x,t\right)}{dx^{2}}-V\left(x\right)\psi \left(x,t\right)=0,$$

which can be written in the Hamiltonian form as

(56) $$iћ\frac{d}{dt}a=\hat{H}a,\;\hat{H}=\frac{{\hat{p}}^{2}}{2m}+V\left(x,t\right),$$

with $\hat{p}$ the momentum:

(57) $$\hat{p}=-iћ\frac{d}{dx}$$

In order to be PT-symmetric, the Hamiltonian needs to be invariant under the parity (*P* performs *x* → −*x*, $\hat{p}$ → −$\hat{p}$) and time reversal operators (*T* performs *i* → −*i*, $\hat{p}$ → −$\hat{p}$), consequently requiring

(58) $$V\left(x\right)=V_{r}\left(x\right)+iV_{i}\left(x\right)$$

where *V_r_*(*x*) = *V_r_* (−*x*) (even function) and *V_i_*(*x*) = −*V_i_*(−*x*) (odd function).

The isomorphism between the paraxial equation of diffraction in optics (Equation (54)) and the Schrödinger equation (Equation (55)) (with *z* playing the role of time and *n*(*x*) playing the role of the optical potential) suggests that the optical potential should have the real part (*n*_r_(*x*)) as an even function (*n*_r_(*x*) = *n*_r_(−*x*)) and the imaginary part (*n*_i_(*x*)) as an odd function (*n*_i_(*x*) = −*n*_i_(−*x*)).

### 9.2. Non-Hermitian Hamiltonians with Optical Waveguides

The isomorphism between the paraxial equation of diffraction in optics and the Schrödinger equation suggests that, in order to have a parity-time-symmetric Hamiltonian, it is sufficient to have the real part of the refractive index as an even function and its imaginary part as an odd function. According to this result, two parallel optical waveguides with the same real part of the refractive index and with opposite imaginary parts realize a PT-symmetric Hamiltonian.

An easy way to verify this is to use the coupled mode theory. In the weak coupling approximation (and neglecting self-coupling), and denoting with *b*_1_ and *b*_2_ the mode amplitudes in two adjacent waveguides, we have [70] (with the implicit time dependence exp(*i*ωt))

(59) $$i\frac{db_{1}}{\mathrm{dz}}=\beta_{1}b_{1}+\kappa_{12}b_{2},$$

(60) $$i\frac{db_{2}}{\mathrm{dz}}=\beta_{2}b_{2}+\kappa_{21}b_{1},$$

where β_1_, β_2_ are the propagation constants of the modes *b*_1_ and *b*_2_, respectively, κ_12_, κ_21_ are the coupling coefficients between the two waveguides, and *z* is the propagation direction. Equations (59) and (60) can be rewritten as

(61) $$i\frac{db}{\mathrm{dz}}=\hat{H}b,$$

with

(62) $$\hat{H}=\left[\begin{array}{cc} \beta_{1} & \kappa_{12}\\ \kappa_{21} & \beta_{2} \end{array}\right].$$

**Figure 7:**
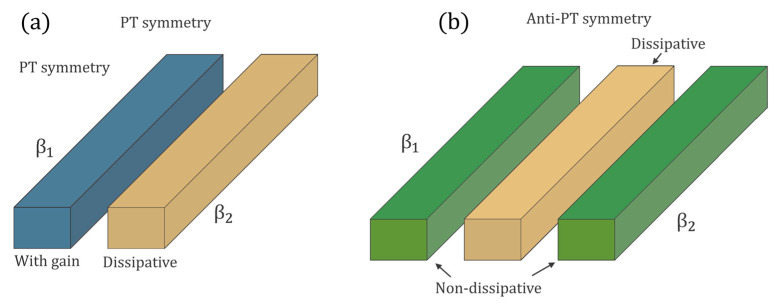
Coupled optical waveguides realizing PT-symmetric (**a**) and anti-PT-symmetric (**b**) Hamiltonians.

So, it is possible to make it PT symmetric using complex propagation constants (considering the effect of gain and loss). In particular, Figure 7a shows that is possible to set up PT-symmetric waveguides by using two directly coupled optical waveguides, one with gain and the other one lossy.

In [27], it has been demonstrated that coupling two optical waveguides to realize an effective anti-PT-symmetric Hamiltonian is also possible using a central auxiliary dissipative waveguide. In particular, considering the coupled mode theory for three coupled waveguides (as in Figure 7b), it is possible to obtain

(63) $$i\frac{db_{1}}{\mathrm{dz}}=\beta_{1}b_{1}+\kappa_{1}c$$

(64) $$i\frac{db_{2}}{\mathrm{dz}}=\beta_{2}b_{2}+\kappa_{2}c$$

(65) $$i\frac{dc}{dz}=\beta_{c}c-i\gamma c+\kappa_{1}^{*}b_{1}+\kappa_{2}^{*}b_{2}$$

where *c* is the mode amplitude in the central waveguide, γ represents the loss rate in a central auxiliary waveguide, and κ_1_ and κ_2_ are the coupling strengths between the external waveguides and the central one.

In [27], the authors demonstrated that in the hypothesis of κ_1_ ≈ κ_2_ and γ << |κ|, mode *c* can be adiabatically eliminated, and an effective anti-PT-symmetric Hamiltonian can be obtained:

(66) $$i\frac{d}{\mathrm{dz}}\left(\begin{array}{c} b_{1}\\ b_{2} \end{array}\right)=\left(\begin{array}{cc} \beta +\Delta -i\Gamma & -i\Gamma \\ -i\Gamma & \beta -\Delta -i\Gamma \end{array}\right)\left(\begin{array}{c} b_{1}\\ b_{2} \end{array}\right)$$

where β = (β_1_+ β_2_)/2, Δ = (β_1_ − β_2_)/2, and Γ = |κ|^2^/γ.

Figure 7b shows the implementation of anti-PT symmetry by means of an auxiliary intermediate dissipative waveguide, making it possible to obtain an imaginary coupling strength.

Recently, an experimental demonstration of anti-PT-symmetric optical waveguides has been reported in [71].

### 9.3. Effective Non-Hermitian Hamiltonians with Optical Resonators

We have demonstrated that the refractive index acts as an optical potential. So, having the real part of the distribution of the refractive index as an even function and the imaginary part as an odd function, gives rise to a PT-symmetric Hamiltonian.

There is a simple way to study PT-symmetric optical resonators using the coupled mode theory proposed in [72]. In particular, a useful formalism to study energy exchanges between the optical resonators was proposed, with a time-domain coupled-mode theory, typical of electronic circuits. Two evanescently coupled optical resonators can be described in the time domain as [72]

(67) $$i\frac{da_{1}}{dt}=-\omega_{1}a_{1}-i\gamma_{1}a_{1}+ka_{2}$$

(68) $$i\frac{da_{2}}{dt}=-\omega_{2}a_{2}-i\gamma_{2}a_{2}+ka_{1}$$

where *a*_1(2)_ represents the energy amplitude in the first (second) cavity, normalized so that |*a*_1(2)_|^2^ is the total energy stored in the first (second) resonator, and ω_1(2)_ and γ_1(2)_ are the resonance angular frequency and the photon decay rate, respectively, of the first (second) resonator and *k* is coupling strength between the resonators.

These two equations can be particularized in two special cases:

- ω_1_ = ω_2_ = ω_0_, γ_1_ = −γ_2_ = γ with *k* a real value (*k* = κ);
- ω_1_ ≠ ω_2_, γ_1_ = γ_2_, with *k* an imaginary value (*k* = *i*κ).

The first case corresponds to an effective PT-symmetric Hamiltonian, whereas the second case becomes anti-PT-symmetric.

**Figure 8:**
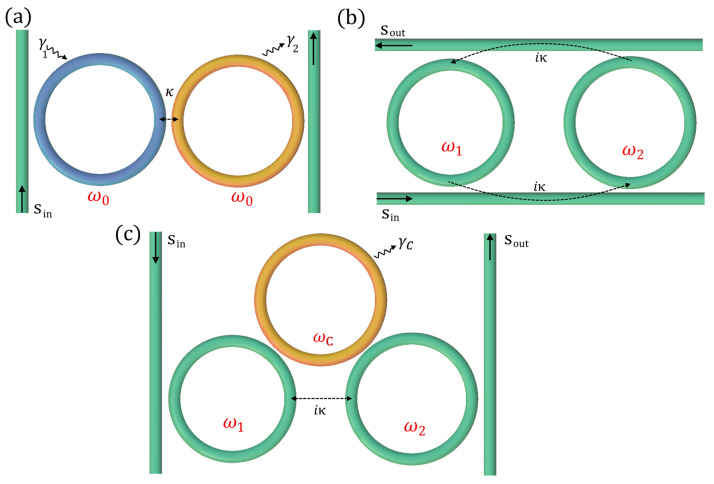
Coupled optical resonators realizing PT-symmetric (**a**) and anti-PT-symmetric (**b**,**c**) Hamiltonians.

The PT-symmetric configuration can be easily realized by directly coupling two optical resonators (Figure 8a), whereas the anti-PT-configurations can be realized by indirectly dissipative coupling (with the same adiabatic approximation for anti-PT-symmetric waveguides), as shown in Figure 8b,c.

### 9.4. Non-Hermitian Sensing on Photonic Integrated Chips

The high sensitivity exhibited by EPs makes it possible to realize high-performance miniaturized sensors; that is the reason for the high interest in non-Hermitian photonics, especially with photonic integrated chips (PICs). There are some works in free-space optics [73], or those with fibre optics [74,75], related to non-Hermitian Hamiltonians, but the high interest in sensitivity enhancement is mainly oriented to the on-chip integration of the sensors. Both the non-resonant parallel-waveguided configuration and the ring-resonant one can be easily integrated in a PIC, to realize a miniaturized sensor for different applications.

In the literature regarding sensing applications, the non-Hermitian configurations realized with waveguided optical resonators (Figure 8) are preferred to the non-resonant waveguide-based ones (Figure 7). The eigenfrequencies of a non-Hermitian photonic system based on resonant cavities can be evaluated by simply measuring the frequencies of the peaks in the transfer function (see Figure 5). Experimentally, the transfer function versus the wavelength can be obtained in three different ways. The first solution requires a broadband source (either integrated or external) and a highly selective spectrometer (either integrated or external) to reconstruct the output spectrum. In the second case a tuneable narrow laser source (integrated or external) is used to scan the spectrum and a photodetector is used to collect the optical power and reconstruct the transfer function versus the wavelength.

With the third method, a broadband source can be used to excite both resonant peaks corresponding to the eigenfrequencies of the non-Hermitian system (in case of real splitting between the eigenfrequencies). In this way, a single photodetector at the output would read the beating between the resonance peaks. So, the Fourier transform of the photogenerated current would show a resonant peak at a frequency equal to the difference between the eigenfrequencies of the non-Hermitian sensor, making the readout really simple.

**Figure 9:**
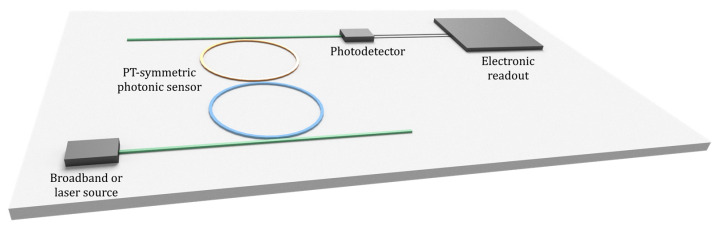
Chip implementation of a PT-symmetric photonic sensor with an integrated source (broadband or laser), a photodetector, and the electronic readout.

All three of the mentioned methods require an electronic readout that can be either integrated on the chip or external (Figure 9).

As seen, PT symmetry strictly requires gain, so a platform providing active materials is needed, as provided with indium phosphide (InP) or gallium arsenide (GaAs). On the contrary, anti-PT symmetry and quasi-PT symmetry can be realized with a fully passive platform such as silicon on insulator (SOI).

## 10. Non-Hermitian Electronics

### 10.1. PT Symmetric Electronics Oscillators

**Figure 10:**
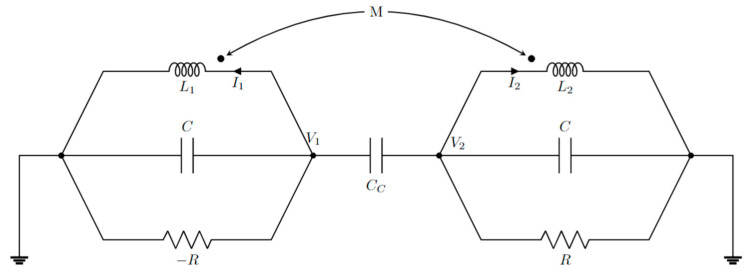
PT-symmetric electronic configuration realized with two coupled RLC resonators (proposed in [13]).

**Figure 11:**
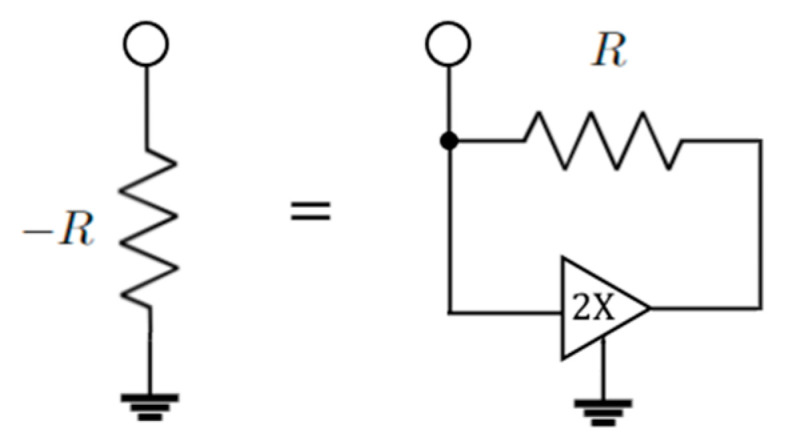
Realization of the negative resistor with a resistor and a 2× amplifier.

Parity-time symmetry can be achieved also with electronics oscillators. Intuitively, by coupling two electronic oscillators, it is possible to realize Hamiltonians equivalent to the ones realized in optics. Figure 10 shows a possible implementation of a PT-symmetric Hamiltonian, implementing two resonators coupled with a capacitance *C_C_*. One of the resonators includes gain thanks to the presence of the negative resistance. A ground referenced negative resistance can be easily realized with a resistor and an amplifier [13] (Figure 11).

Considering Figure 10, using the Kirchhoff laws for voltages and currents, we obtain

(69) $$V_{1}=i\omega \left(LI_{1}+MI_{2}\right)$$

(70) $$I_{1}-\frac{V_{1}}{R}+i\omega CV_{1}+i\omega C_{c}\left(V_{1}-V_{2}\right)=0$$

and

(71) $$V_{2}=i\omega \left(LI_{2}+MI_{1}\right)$$

(72) $$I_{2}+\frac{V_{2}}{R}+i\omega CV_{2}+i\omega C_{c}\left(V_{2}-V_{1}\right)=0$$

Combining Equations (69)–(72) it is possible to arrive at

(73) $$\left(\begin{array}{cc} \frac{L}{i\omega \left(L^{2}-M^{2}\right)}-\frac{1}{R}+i\omega \left(C+C_{c}\right) & -i\omega C_{c}-\frac{M}{i\omega \left(L^{2}-M^{2}\right)}\\ -i\omega C_{c}-\frac{M}{i\omega \left(L^{2}-M^{2}\right)} & \frac{L}{i\omega \left(L^{2}-M^{2}\right)}+\frac{1}{R}+i\omega \left(C+C_{c}\right) \end{array}\right)\left(\begin{array}{c} V_{1}\\ V_{2} \end{array}\right)=0$$

Defining *k* = *M*/*L*, in the hypothesis of *k* << 1 and approximating ω (ω_n_/ω^2^ − 1)/2 ≈ ω_n_ − ω, it is possible to arrive at

(74) $$\left(\begin{array}{cc} i\left(\omega_{0}-\omega \right)+\gamma & -i\kappa \\ -i\kappa & i\left(\omega_{0}-\omega \right)-\gamma \end{array}\right)\left(\begin{array}{c} V_{1}\\ V_{2} \end{array}\right)=0$$

with

(75) $$\kappa =\left(\frac{k\omega_{0}{}^{2}}{2\omega }-\frac{\omega C_{c}}{2\left(C+C_{c}\right)}\right)$$

(76) $$\gamma =\frac{1}{2R\left(C+C_{c}\right)}\;$$

(77) $$\omega_{0}=\frac{1}{L\left(C+C_{c}\right)}\;$$

For time-harmonic voltages, Equation (74) is equivalent to the Schrödinger equation with a PT-symmetric Hamiltonian. So, it is possible to create a PT-symmetric electronic system by coupling two resonators with opposite gains by means of a coupling capacitance or a mutual inductance (or both).

### 10.2. Anti-PT-Symmetric Electronic Oscillators

**Figure 12:**
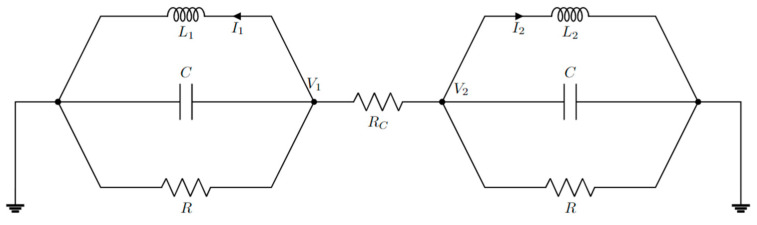
Anti-PT-symmetric electronic configuration realized with two coupled RLC resonators (architecture proposed in [76]).

Figure 12 schematizes the idea of two coupled resonators for anti-PT symmetry proposed in [76].

Combining Kirchhoff’s laws, it is possible to obtain

(78) $$\frac{V_{1}}{i\omega L_{1}}+\frac{V_{1}}{R}+i\omega C_{1}V_{1}+\frac{1}{R_{C}}\left(V_{1}-V_{2}\right)=0$$

(79) $$\frac{V_{2}}{i\omega L_{2}}+\frac{V_{2}}{R}+i\omega C_{2}V_{2}+\frac{1}{R_{C}}\left(V_{2}-V_{1}\right)=0$$

Combining them in a matrix form:

(80) $$\left(\begin{array}{cc} \frac{1}{i\omega L_{1}}+\left(\frac{1}{R}+\frac{1}{R_{C}}\right)+i\omega C & -\frac{1}{R_{C}}\\ -\frac{1}{R_{C}} & \frac{1}{i\omega L_{2}}+\left(\frac{1}{R}+\frac{1}{R_{C}}\right)+i\omega C \end{array}\right)\left(\begin{array}{c} V_{1}\\ V_{2} \end{array}\right)=0.$$

For *R* = *R_C_*, we obtain

(81) $$\left(\begin{array}{cc} i\left(\omega_{1}-\omega \right)-\gamma & \kappa \\ \kappa & i\left(\omega_{2}-\omega \right)-\gamma \end{array}\right)\left(\begin{array}{c} V_{1}\\ V_{2} \end{array}\right)=0,$$

where

(82) $$\kappa =\frac{1}{2R_{C}C}$$

(83) $$\omega_{n}=1/\sqrt{L_{n}C}$$

(84) $$\gamma =\left(\frac{1}{R}+\frac{1}{R_{C}}\right)\frac{1}{C}.$$

For time-harmonic voltages, Equation (81) is equivalent to the Schrödinger equation with an anti-PT-symmetric Hamiltonian. So, it is possible to realize anti-PT-symmetry with two electronic resonators coupled with a resistor.

### 10.3. Non-Hermitian Sensing with Electronic Boards

**Figure 13:**
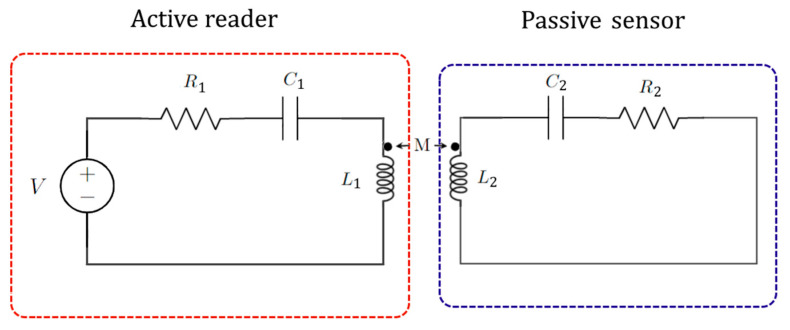
Generic implementation of coupled circuits for telemetry that can be particularized for non-Hermitian electronic sensing.

The majority of non-Hermitian sensors in electronics has been developed on printed circuit boards (PCBs) using discrete components soldered onto it. A lot of interest has been shown for non-Hermitian telemetry: two electronic systems (one active reader realized with a PCB or chip, including a source and a resonator, and the other a passive electronic resonator) communicate wirelessly between each other, thanks to the mutual coupling between inductors (see Figure 13) (the passive sensor can be even implanted under the skin for biological sensing [77]). In this way a non-Hermitian Hamiltonian is realized and the perturbation to the sensor or to the mutual coupling between the resonators is enhanced, thus realizing high sensitivity.

## 11. Sensing Applications of Non-Hermitian Photonics

In this section, some of recent sensing schemes applied in photonics will be shown. The two most studied applications of non-Hermitian sensing in photonics are optical gyroscopes and particle sensors.

### 11.1. Non-Hermitian Optical Gyroscopes

In [78], Ren et al. proposed for the first time a PT-symmetric optical gyroscope. A gyroscope is a sensor able to measure the angular velocity of its frame with respect to an inertial system. According to the Sagnac effect, the resonance frequency shift of a single isolated rotating optical ring resonator with respect to a rest condition is [79]

(85) $${\Delta \omega }_{\Omega ,i}=\pm \frac{2\pi R_{i}\Omega }{\lambda n_{\mathrm{eff}}},$$

where λ is the wavelength in vacuum, *R*_*i*_ the radius of the *i*-th ring resonator, *n*_eff_ is the effective index of the optical waveguides and Ω is the angular velocity of the frame. The minus or plus sign is chosen if the mechanical rotation is in the same or opposite sense, respectively, of the rotation of the optical beam in the resonator.

**Figure 14:**
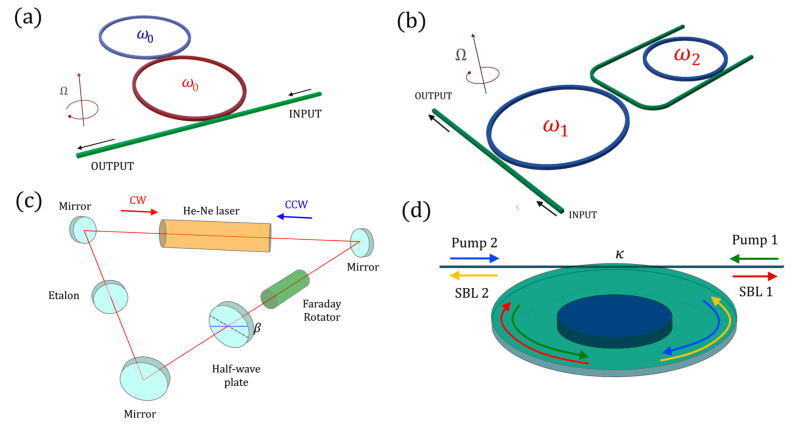
(**a**) Schematic of a parity-time-symmetric laser gyroscope system (proposed in [78]). (**b**) Schematic of an anti-parity-time-symmetric gyroscope (proposed in [81]). (**c**) Non-Hermitian ring laser gyroscope (proposed in [73]). (**d**) Brillouin laser gyroscope at the EP (proposed in [83]).

The PT-symmetric gyroscope presented in [78] is based on the standard PT-symmetric structure realized with two coupled resonators with perfectly balanced gain and loss (Figure 14a) and with the same radius.

A splitting between the real part of the eigenfrequencies has been demonstrated to be

(86) $${\Delta \omega }_{PT}\approx 2\sqrt{\left|{\Delta \omega }_{\Omega ,i}\kappa \right|}$$

where κ is the coupling strength between the cavities. Since the coupling strength is inversely proportional to the radius of the ring resonators, the spectral splitting is independent of the size of the device. This explains the wide interest in research for non-Hermitian gyroscopes. The authors demonstrated that the gyroscope exhibits a sensitivity enhancement with respect to the resonance frequency shift in a single ring, equal to

(87) $$\frac{{\Delta \omega }_{PT}}{{\Delta \omega }_{\Omega ,i}}=\sqrt{\left|2\kappa /{\Delta \omega }_{\Omega ,i}\right|}.$$

In [80] the PT-symmetric gyroscope has been theoretically studied, introducing doubts about the existence of a measurable splitting on the output transfer function of the sensor, due to the complex splitting between eigenfrequencies during rotation. Later, anti-PT-symmetric versions of the optical gyroscope were proposed to overcome the problems of instability illustrated in Section 5 (Figure 14b): in [81], a U-shaped waveguide was used to indirectly couple two resonators, whereas in [82] a single bus between the resonators was proposed as a more stable solution for realizing the anti-PT-symmetric gyroscope, with a proposal of integrating this solution in the InP platform.

In [73], a ring laser gyroscope was set up in the proximity of an EP (Figure 14c). The device was realized in free space by inserting a Faraday rotator and a half-wave plate inside the optical resonator, realizing a non-reciprocal loss for the counterpropagating optical modes, clockwise (CW) and counterclockwise (CCW). According to experimental results, an enhancement factor 20 was obtained for the resonance splitting in the vicinity of an EP.

In [83], a very high quality microdisk is used to realize a new kind of non-Hermitian gyroscope (Figure 14d). The stimulated Brillouin effect leads to the lasing of counterpropagating modes in the microdisk, with ultranarrow linewidths. Moreover, the Brillouin effect perturbs the resonating frequencies even in the absence of rotation, thus leading to an effective anti-PT-symmetric Hamiltonian.

By adjusting the pump frequency, it is possible to reach an EP. The angular velocity, Ω, leads to a perturbation of the Hamiltonian of the system. Experimental results demonstrated the expected enhancement in the spectral response of the gyroscope by a factor of 4.

### 11.2. Particle Sensing: Exceptional Points and Exceptional Surfaces

**Figure 15:**
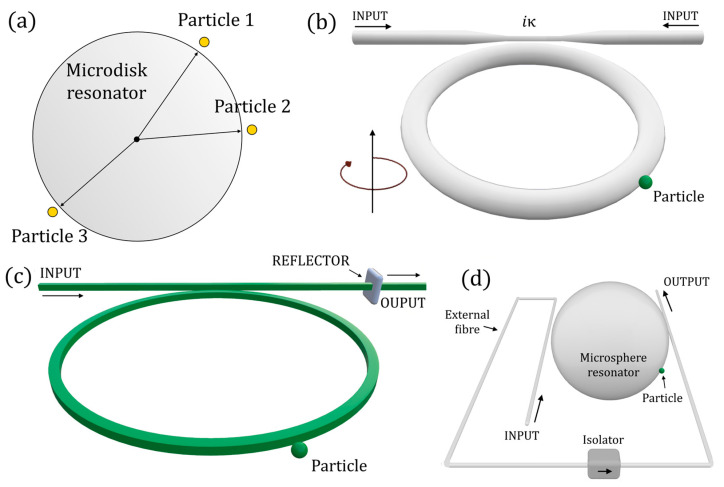
(**a**) Schematic of the microdisk resonator for particle sensing at an EP (two particles realize the EP and the third one is sensed) (proposed in [84]). (**b**) Schematic of an anti-PT-symmetric particle sensor exploiting rotation to reach the EP (architecture proposed in [87]). (**c**) Schematic of the exceptional surface configuration for particle sensing proposed in [64]. (**d**) Schematic of the implementation of a microsphere resonator for particle sensing at the exceptional surface: the non-reciprocal coupling between counterpropagating modes is realized via an optical isolator in the external coupling fibre (architecture proposed in [68]).

Another highly investigated application of non-Hermitian photonics is particle sensing. In [84], Wiersig demonstrated the possibility of applying EPs to single nanoparticle sensing (Figure 15a). The effective Hamiltonian for the microdisk with *N* nanoparticles in the travelling-wave basis (CCW, CW) is given by (adapted to the time-harmonic convention here adopted)

(88) $${\hat{H}}^{\left(N\right)}=\left(\begin{array}{cc} -\Omega^{\left(N\right)} & A^{\left(N\right)}\\ B^{\left(N\right)} & \;-\Omega^{\left(N\right)} \end{array}\right).$$

with

(89) $$\Omega^{\left(N\right)}=\Omega^{\left(0\right)}+\overset{N}{\underset{j=1}{\sum }}\left(V_{j}+U_{j}\right)$$

(90) $$A^{\left(N\right)}=\overset{N}{\underset{j=1}{\sum }}\left(V_{j}-U_{j}\right)e^{-i2m\beta_{j}}$$

(91) $$B^{\left(N\right)}=\overset{N}{\underset{j=1}{\sum }}\left(V_{j}-U_{j}\right)e^{i2m\beta_{j}}.$$

The quantities 2*V_k_* and 2*U_j_* represent the complex frequency shifts for the positive and negative parity modes due to particle *j* alone.

Wiersig proposed a system with three particles, two of them generating the EP, and the third one being the perturbing one. A diabolic point is realized when *B*^(2)^ = *A*^(2)^ = 0 (no scattering between CW and CCW travelling waves), whereas an EP results in *B*^(2)^ = 0 or *A*^(2)^ = 0; this principle is behind what has later been introduced as an exceptional surface, because the use of a single optical cavity ensures a hypersurface of EP.

Wiersig demonstrated that in the presence of the third particle, at a diabolic point, the induced complex frequency splitting is given by

(92) $${\Delta \Omega }_{DP}=2\left(V_{3}-U_{3}\right).$$

Instead, in the presence of an EP, the splitting is given by (for *B*^(2)^=0)

(93) $${\Delta \Omega }_{\mathrm{EP}}={\Delta \Omega }_{\mathrm{DP}}\sqrt{1+\frac{A^{\left(2\right)}e^{i2m\beta }}{V_{3}-U_{3}}}.$$

If the square root is larger than one, the splitting at the EP is enhanced.

In [85], the enhancement of the splitting predicted by Wiersig was experimentally demonstrated in an optical microcavity. In a log–log graph, the slope of the splitting with respect to the perturbation equal to 1/2 was demonstrated, being different from the slope of 1 at the diabolic point.

Later, in [86], an anti-PT-symmetric device was proposed for particle sensing. In [87], a spinning resonator (rotating around its centre) was proposed for reaching the EP in an anti-parity-time-symmetric system, realized with a single cavity, for ultrasensitive nanoparticle sensing (see Figure 15b). In particular, the rotation induces a difference between the two resonances of the counterpropagating modes (see Equation (85)), thus making it possible to obtain an anti-PT-symmetric Hamiltonian. The indirect coupling mechanism necessary for the anti-PT-symmetric configuration is realized with an external fibre, implementing an optical isolator.

In [88], a whispering gallery mode parity-time-symmetric nanoparticle sensor has been proposed. The presence of gain in the PT-symmetric configuration allows the narrowing of the linewidths, helping to increase the resolution, thus improving the limit of detection of nanoparticles.

In [64], the concept of ES was introduced for the first time by Zhong et al. The idea proposed by the authors was to exploit the EPs to enhance the sensitivity of a particle sensor, without being subject to undesired perturbations. The solution was implemented with the architecture in Figure 15c.

A scattering matrix method was used to derive the transfer function and the difference between the eigenvalues *ϕ* was obtained:

(94) $$\Delta \phi =2\sqrt{r_{p}^{2}+r_{m}^{\prime }\eta^{2}r_{p}},$$

where *r*_p_ is the amplitude reflection of the particle to be sensed, $r_{m}^{\prime }$ the effective unidirectional coupling from CW mode to CCW mode, and η^2^ is the nondimensional power coupling coefficient between the waveguide and ring resonator.

For very small values of *r*_p_, the splitting between the eigenvalues becomes

(95) $$\Delta \phi_{\mathrm{EP}}\approx \{\begin{array}{cc} 2\eta \sqrt{r_{p}}, & r_{p}\ll \eta^{2}\\ 2r_{p}, & r_{p}\gg \eta^{2} \end{array}.$$

For *r*_p_ << η^2^, the splitting is proportional to the square root of the perturbation, *r*_p_, in perfect agreement with the condition of the EPs. The advantage of the proposed solution is that any undesired perturbation to the cavity does not affect the condition of the EP, making the entire system much more robust than classical EP-based sensors.

In [68], an integrated ES-based particle sensor has been experimentally realized, demonstrating the enhancement in the frequency splitting caused by small perturbations. The non-reciprocal coupling between counterpropagating modes CW and CCW in a silica microsphere was realized with an optical tapered fibre coupled twice from two sides of the microsphere (see in Figure 15d): the presence of a fibre-based optical isolator in the coupling fibre realized the nonreciprocal coupling between the counterpropagating modes.

In [89], a nonreciprocal coupling between the counterpropagating optical modes in a single optical resonator is proposed to minimize the detection limit, thanks to the fact that the two optical modes do not degenerate at the EP.

### 11.3. Other Sensing Applications of Non-Hermitian Optics

**Figure 16:**
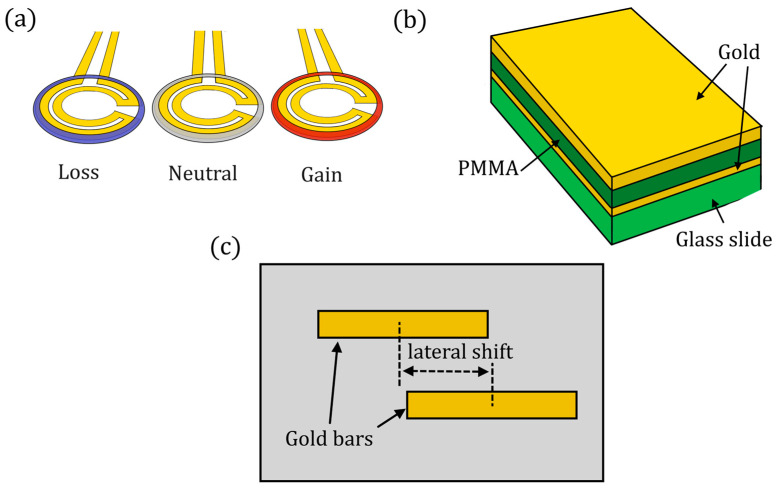
(**a**) A parity-time-symmetric ternary micro-ring system with equidistantly spaced cavities (proposed in [90]). (**b**) Schematic drawing of the thermo-sensitive glass slide engineered at an EP. A three-layer structure of Au–PMMA–Au is deposited on a silica glass slide (proposed in [91]). (**c**) One of the configurations of the plasmonic structure (repeated periodically) made of two optically dissimilar plasmonic resonators array with detuned resonances. The detuning can be implemented either using structures of distinct size or using identical resonators in distinct optical environments (architecture proposed in [94]).

There are several other applications of non-Hermitian optics. In [90], a higher-order EP has been experimentally demonstrated. A cube root dependence on induced perturbations in the refractive index was shown thanks to the coupling between three resonators. A 3 × 3 Hamiltonian was used to model the device, in which one resonator is lossy, another has gain, and the central one is neutral (see Figure 16a):

(96) $$\hat{H}=\left(\begin{array}{ccc} ig+\varepsilon & \kappa & 0\\ \kappa & 0 & \kappa \\ 0 & \kappa & -ig \end{array}\right).$$

in which +*g* or −*g* accounts for the gain or loss, respectively. This kind of Hamiltonian shows a dependence on the perturbation as ε^1/3^.

In 2018, Zhao et al. proposed a coating of an optical EP structure for thermal sensing with a fine spatial resolution [91]. In particular, a three-layer structure of Au–PMMA–Au was deposited on a silica glass slide for engineering the thermo-sensitive glass slide at an EP (Figure 16b).

In 2019, the EP of an optomechanical cavity was exploited to enhance the sensitivity of a mass sensor [92]. The gain or loss was engineered by driving the cavity with a blue-detuned or red-detuned laser, respectively.

A magnetometer with exceptional sensitivity, using cavity magnon polaritons with PT symmetry, was proposed in [93]. A third-order EP leads to an estimated magnetic sensitivity of 10^−15^ T Hz^−1/2^ in the strong coupling region, which is two orders of magnitude higher than that of the state-of-the-art magnetoelectric sensor.

In [94], plasmonic EPs are demonstrated, which are based on the hybridization of detuned resonances in multilayered plasmonic structures. The reaching of a critical complex coupling rate between nanoantenna arrays (Figure 16c shows one of the proposed configurations) results in the simultaneous coalescence of the resonances and loss rates, thus allowing the reaching of the EP. This setup is proposed for sensing of anti-immunoglobulin G, the most abundant immunoglobulin isotype in human serum.

In 2020, an ultrasensitive stress sensor was proposed [95], with parity-time-symmetric cavities. In particular, one cavity is embedded on a cantilever beam, serving as the sensing element. The authors claim a sensitivity enhancement of about 8 orders of magnitude at a stress range between 0 and 1 nPa.

In [96], it has been demonstrated with a Brillouin microresonator that two nondegenerate EPs behave anisotropically; i.e., when approached from both directions, the sensitivities to the deviations of the two supermodes function differently. This has been proposed to be used for realizing a bi-scale supersensitive optical sensor that can detect particles of different sizes at the same time.

In [97], a label-free biosensor for detecting low-concentration analytes has been proposed, via coupled resonant optical-tunnelling resonators (one lossy cavity and one sensing cavity). The behaviour around the EP is controlled by adjusting the separation between the resonators (thus the coupling strength). The surface of the sensing cavity is biofunctionalized in advance to bind specific target analytes, which perturb the EP causing an additional absorption in the sensing cavity. The authors evaluated the effect of the presence of the analyte as a change in the imaginary part unit of refractive index (IP): a sensitivity of 17.12 nm/IP was demonstrated, with a detection limit of 4.2 × 10^−8^ IP, corresponding to 1.78 ng for sensing of carcino-embryonic antigen (CEA).

A gas sensor with ultrahigh sensitivity has been shown in [98]: the transverse displacement induced by the photonic spin Hall effect (PSHE) is sensitive to the variation in refractive index in gas media, especially in the proximity of an EP. The sensitivity of the gas sensing can reach 10^−6^ RIU µm^−1^, if the in-plane wavevector component of the probe Gaussian light is reduced.

In [56], coupled cavities at the EP have been studied for refractive index and absorption sensing.

In [74], two counterpropagating modes in a fibre-ring cavity with different losses were used to enhance the sensitivity of a fibre sensor. The differential roundtrip loss is induced by using an extra fibre ring with an optical isolator. An erbium-doped fibre is used to narrow the linewidth (pushing the eigenfrequencies near to the real axis).

## 12. Sensing Applications of Non-Hermitian Electronics

Non-Hermitian sensing has been exploited also in electronics for different kinds of sensing. Here, some recent outstanding advances in non-Hermitian sensing in electronics will be illustrated.

### 12.1. Generalized PT Symmetry for Enhanced Sensor Telemetry

A generalized condition for PT symmetry has been shown in [99]. In particular, the theory of the so-called isospectral parity-time reciprocal scaling (PTX) symmetry has been developed.

As shown, PT symmetry is achieved with perfectly balanced gain and loss, corresponding to the negative and positive resistors in the coupled-oscillators electric system. This results in sharp and deep resonances, with improved spectral resolution and modulation depth for sensing. Sometimes, however, practical implementations for the sensor telemetry may encounter difficulties in achieving an exact conjugate impedance profile [99], due, for example, to space limitations: when using miniaturized MEMS implanted sensors, the inductance of the sensor’s microcoil, *L*_S_, is usually smaller than the one of the reader’s coil, *L*_R_. In principle, downscaling the reader coil can match *L*_R_ to *L*_S_; however, this would lead to a reduced mutual inductive coupling and degrade the performance of the sensor.

This is why the authors introduced an extra degree of freedom to enable the arbitrary scaling of the coil inductance and other parameters, to improve the wireless interrogation.

**Figure 17:**
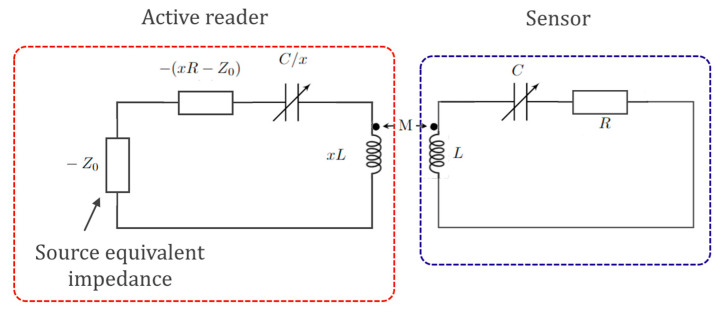
Equivalent circuit model for the PTX-symmetric telemetric sensor. The active reader interrogates the sensor via magnetic coupling. The parameter *x* is the scaling parameter. For *x* = 1, the PTX degenerates into PT symmetry (architecture proposed in [99]). The source is modelled via an equivalent impedance −Z_0_. The voltage impedance-controlled converter.

The added degree of freedom is the parameter *x*, in the PTX configuration shown in Figure 17, where *x* is the scaling factor of the PTX symmetry: for *x* = 1, the system degenerates in the classical electronic PT-symmetric circuit.

### 12.2. Implantable Microsensors

**Figure 18:**
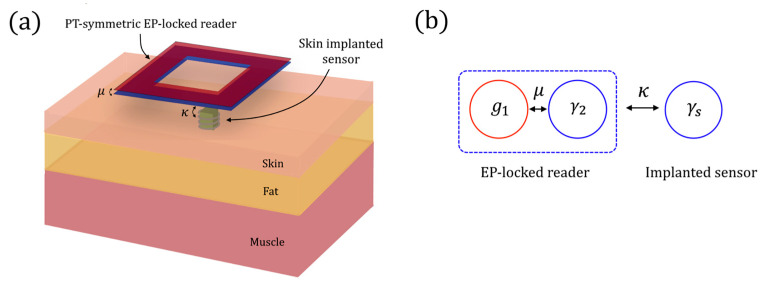
(**a**) Schematic of the EP realized with the implantable LC sensor and the combination of external cavities with gain and loss (proposed in [77]). (**b**) Architecture of an EP-locked reader proposed in [77].

In biomedicine, some implanted electronic sensors are based on resonant inductor-capacitor (LC) circuits that monitors internal physiological states. However, the sensitivity of the wireless interrogation technique is often low, thus limiting the possibility of realizing minimally invasive devices for continuous physiological monitoring. In [77], the authors proposed the readout of an implantable microsensors using a wireless system locked to an EP. The coupling strength *κ* between the implanted sensor (see Figure 18a) and the reader represents the parameter to be sensed. The idea of using the passive LC circuit as the lossy part of a PT-symmetric sensor is not the best choice for this kind of setup, because the sensitivity of a PT-symmetric device is null for κ = 0 (see Figure 18b).

The authors demonstrated that the spectral response, Δω, of the reader biased at an EP follows a dependency of Δω ∝ *κ*^2/3^, which greatly amplifies its response to a weakly coupled sensor.

The coupled mode equations describing the combination of the sensor and the PT-symmetric reader are given by

(97) $$\frac{d}{dt}\left(\begin{array}{c} a_{1}\\ a_{2}\\ a_{s} \end{array}\right)=\left(\begin{array}{ccc} i\omega_{1}+g_{1} & -i\mu & -i\kappa \\ -i\mu & i\omega_{2}-\gamma_{2} & -i\kappa \\ -i\kappa & -i\kappa & i\omega_{s}-\gamma_{s} \end{array}\right)\left(\begin{array}{c} a_{1}\\ a_{2}\\ a_{s} \end{array}\right),$$

where subscript 1 and 2 refer to the gain resonator and the loss resonator of the PT symmetric sensor, and subscript *s* refers to the implanted sensor resonator. The term *a_j_* (with *j* = 1, 2, *s*) represents the mode amplitudes and ω*_j_* (with *j* = 1, 2, *s*) the resonant frequencies of the three resonators. Moreover, *g*_1_ indicates the gain rate of the first resonator, γ_2_ indicates the loss rate of the second resonator of the reader, γ_s_ indicates the loss rate of the sensor resonator, and µ is the coupling strength between the two resonators of the reader. To be PT symmetric, the reader requires ω_1_ = ω_2_ = ω_0_, and *g*_1_ = γ_2_.

With the Newton–Puiseux series, the authors found the eigenfrequencies of the system depart from the central frequency ω_0_, with a dependence proportional to *κ*^2/3^.

This configuration has been experimentally proven to have a sensitivity 3.2 times the limit encountered by existing schemes.

### 12.3. Non-Hermitian Accelerometer

**Figure 19:**
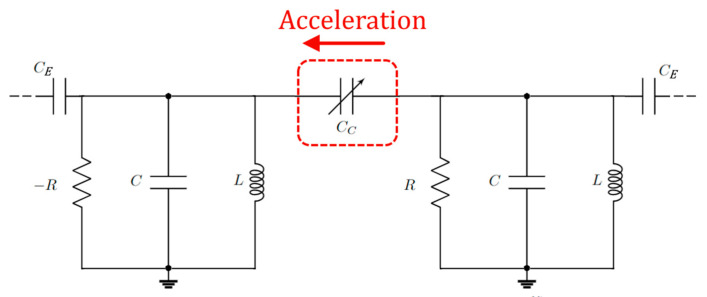
Schematic of the PT-symmetric electromechanical accelerometer proposed in [100]. The PT-symmetric circuit coupled to the transmission line with capacitors *C_E_*. The capacitance *C_C_* realizes the coupling between the two RLC resonators.

In [100], a preprint, an electromechanical accelerometer is demonstrated; it is a variable capacitor, *C_C_*, with one plate connected to a test mass that senses the acceleration. The electrical scheme is represented in Figure 19. The authors demonstrated that, thanks to the coupling *C_E_*, the noise, due to collapse of the eigenvectors (demonstrated for the Brillouin gyroscope in [62]), is mitigated, exploiting the detuning from a transmission peak degeneracy (TPD) when the sensor is weakly coupled to transmission lines. The device shows a three-fold signal-to-noise enhancement with respect to configurations working away from TPD.

In particular, the authors introduce a normalized Hamiltonian for the PT-symmetric isolated system (in the absence of *C_E_*) (here adapted to the chosen convention for the time-harmonic evolution):

(98) $${\tilde{H}}_{0}\equiv \frac{{\hat{H}}_{0}}{\omega_{0}}=\left(\begin{array}{cc} -1+i\gamma_{0} & \kappa_{0}\\ \kappa_{0} & -1-i\gamma_{0} \end{array}\right),$$

where γ_0_ is the normalized gain (loss) in each resonator and κ_0_ is the normalized coupling strength between the resonators. As required by the condition of the EP of the PT-symmetric Hamiltonian, κ_0_ = γ_0_. Under this hypothesis, including the effect of perturbation ε (variation of the capacitance, due to acceleration) and the effect of the linewidth broadening (γ_e_) due to the coupling with the transmission line, the effective Hamiltonian is modified into

(99) $${\tilde{H}}_{\mathrm{eff}}=\left(\begin{array}{cc} -1+\varepsilon -i\gamma_{e}+i\gamma_{0} & \gamma_{0}+\varepsilon \\ \gamma_{0}+\varepsilon & -1+\varepsilon -i\gamma_{e}-i\gamma_{0} \end{array}\right).$$

Looking for the transmittance of the system and considering the frequencies associated (ῶ_±_) with the transmission peaks (physical observables, that differ from eigenfrequencies), they obtained

(100) $${\tilde{\omega }}_{\pm }=\{\begin{array}{cc} 1-\varepsilon ; & \varepsilon \leq \varepsilon_{\mathrm{TPD}}\\ 1-\varepsilon \pm \sqrt{2\gamma_{0}\epsilon +\varepsilon^{2}-\gamma_{e}^{2}}; & \varepsilon \geq \varepsilon_{\mathrm{TPD}} \end{array}\;.$$

The frequencies associated to the transmission peaks have a coalescence point at

(101) $$\varepsilon_{\mathrm{TPD}}=-\gamma_{0}+\sqrt{\gamma_{0}^{2}+\gamma_{e}^{2}}\;.$$

So, the maximum sensitivity is around ε = ε_TPD_. Around the transmission peak degeneracy (TPD) point, the bi-orthogonal basis of the effective Hamiltonian does not collapse and the Petermann factor does not diverge. Since the Petermann factor was the source of the sensitivity limitations in the Brillouin ring laser gyroscope in [62], the separation between the coalescence of the eigenmodes and the coalescence of the measurable frequencies associated with the transmission peaks overcomes the limitations of the Petermann factor.

### 12.4. Other Sensing Applications of Non-Hermitian Electronics

In [101], capacitive sensing for different applications (microfluidic flow sensor, pressure sensor, accelerometer) is performed by implementing a sixth-order EP with non-degraded thermal noise performance. A capacitive coupling channel is used as a sensing platform to achieve an enhanced resonance shift proportional to the fourth-order root of the perturbation strength, maintaining a high resolution for weak perturbation. The thermal noise is mitigated to a level comparable to the Hermitian counterpart, despite the highly noisy gain and loss elements.

In [102], an ultra-sensitive passive wireless sensor is demonstrated, exploiting high-order EP for weak coupling detection. In particular, a spectral splitting proportional to the cube root of the coupling between two wirelessly coupled electronic RLC resonators is demonstrated.

In [103], a glucose sensor with enhanced sensitivity has been proposed, using a PT-symmetric system that sandwiches the tissue sample under analysis. The glucose level changes within the skin are sensed by measuring the frequency shift in the electromagnetic resonance induced in the PT-symmetric system. The skin is modelled as a transmission line.

## 13. Conclusions

In this work, recent progress in EP-based sensors has been reviewed, with a particular focus on implementations of non-Hermitian Hamiltonians in optics and electronics. A theoretical overview of the non-Hermitian Hamiltonian is presented, in order to be a helpful starting point for the conceptualization and design of non-Hermitian sensors.

Several experimental works were then shown, demonstrating the real advantage of non-Hermitian sensing with respect to classical sensing principles, in several fields of sensing (especially, but not only, angular velocity and particle sensing in optics and wireless telemetry in electronics).

The debate on the influence of noise on EPs is still open; however, new techniques to avoid the negative effect of noise are now under research. The concept of an exceptional surface has been introduced in optics to make the sensor immune to unwanted external perturbations, and new configurations have been proposed and a design at the transmission peak degeneracy point has been recently introduced in electronics to prevent the coalescence of eigenmodes at the EP, thus improving its robustness.
